# “Could she be a good mother?. The stigma of mental illness in motherhood. A case report

**DOI:** 10.1192/j.eurpsy.2022.1444

**Published:** 2022-09-01

**Authors:** M.V. López Rodrigo, A. Osca Oliver, M. Palomo Monge, M. Pérez Fominaya

**Affiliations:** 1 Hospital Nuestra Señora del Prado, Psiquiatría, Talavera de la Reina, Spain; 2 Hospital Nuestra Señora del Prado, Psiquiatria, Talavera de la Reina, Spain

**Keywords:** stigma, motherhood, mental illness

## Abstract

**Introduction:**

Approximately 15% of pregnant women suffer from a mental illness, however only half of them accept psychopharmacological treatment. One of the reasons for refusing treatment is the stigma attached to it.An important part of functional recovery is defining identity. This identity is multifactorial and is defined by several variables, one of them being gender.Several studies on motherhood in women with mental illness define the importance of a mothering identity, providing meaning and values. We present the case of a 39-year-old woman, mother of a two-year-old child, undergoing follow-up at a psychiatric clinic for recurrent depressive episodes and a history of two suicide attempts ten years ago. Currently stable in treatment with escitalopram 10 mg and lorazepam 1 mg if necessary.The woman refers the desire to abandon treatment after realizing that she is pregnant again. Therapeutic accompaniment is decided. The social worker from the obstetric service communicates with the psychiatric service to question the woman’s ability to care for a child with her psychiatric history.

**Objectives:**

Determine the stigma of mental illness, including among healthcare workers.

**Methods:**

The woman makes her decision with full judgment. The patient is accompanied during pregnancy without incident, with clinical stability.

**Results:**

After delivery, the patient decides to resume psychopharmacological treatment.

**Conclusions:**

Having a mental illness does not determine a woman’s ability to be a mother. As long as it is agreed with the psychiatrist, patients have the right to make decisions about their treatment.

**Disclosure:**

No significant relationships.

